# TRiP stocks contain a previously uncharacterized loss-of-function sevenless allele

**DOI:** 10.17912/micropub.biology.000097

**Published:** 2019-04-04

**Authors:** Spencer E Escobedo, Jonathan Zirin, Vikki M Weake

**Affiliations:** 1 Department of Biochemistry, Purdue University, West Lafayette, IN, 47907; 2 Harvard Medical School, Boston, MA, 02115

**Figure 1.**
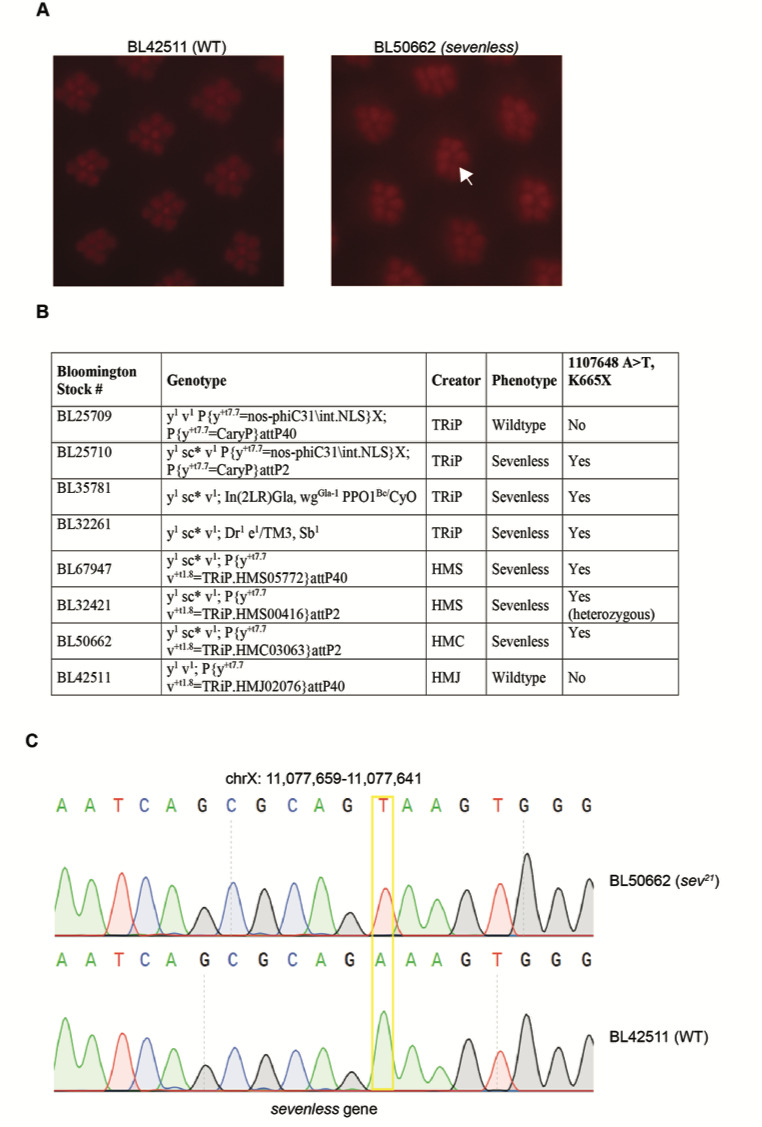
Identification of a new *sevenless (sev)* allele in a subset of TRiP stocks **A** Representative images showing adult retinas imaged using optical neutralization for male flies from the TRiP collection that show wild type (BL42511) or *sev* (BL50662) phenotypes. White arrow indicates expected position of missing R7 rhabdomere. **B** Summary table describing presence of the eye phenotype and *sev* mutation in tested TRiP stocks. Eye phenotypes were analyzed using optical neutralization on male flies. Note, BL35781 and BL32261 females were outcrossed to Oregon R males with *y^1^ sc v^1^; +/CyO* and *y^1^ sc v^1^; +/TM3, Sb^1^* male progeny used to assess presence/absence of R7. **C** Chromatogram showing sequence analysis of the *sev* gene in BL42511 and BL50662 stocks; the position of the 1107648A>T mutation corresponding to the new *sev^21^* allele is shown.

## Description

The Transgenic RNAi Project (TRiP) has generated more than 12,000 transgenic RNAi fly stocks that have been distributed to the community via the Bloomington Drosophila Stock Center (Ni et al. 2007; Ni et al. 2011; Perkins et al. 2015). These stocks express long double-stranded RNA hairpins (dsRNAs) or short RNA hairpins (shRNAs) under GAL4/UAS control (Brand and Perrimon 1993), and provide powerful tools for targeted genetic screens. Unexpectedly, as part of a genetic screen examining retinal degeneration in flies, we identified a defect in eye development associated with many of the TRiP stocks. *Drosophila* have a compound eye composed of repeating units, termed ommatidia, that each contain eight photoreceptor cells (R cells 1 – 8) (Ready, Hansen and Benzer 1976). The light-sensing organelle, the rhabdomere, in seven of these photoreceptors can be directly visualized in wild-type flies using light microscopy either by optical neutralization or by examining the deep pseudopupil; R7/R8 are stacked on top of each other so only one is visible in a given vertical plane (Franceschini and Kirschfeld 1971).Whereas seven rhabdomeres could be counted per ommatidium in wild-type flies (Fig. 1A), a subset of the TRiP lines tested show characteristic loss of a single rhabdomere (Fig. 1A, Fig. 1B). This single photoreceptor loss phenotype is reminiscent of *sevenless* *(sev)* mutants; *sev* (FBgn0003366) encodes a receptor tyrosine kinase essential for development of R7; thus, loss of function *sev* mutations result in ommatidia that lack R7 (Harris et al. 1976; Simon, Bowtell and Rubin 1989). Preliminary observations suggested that the *sev* phenotype was X-linked and observed only in TRiP stocks containing a *scute (sc)* allele of unknown origin denoted sc^*^. Whole genome sequencing data for one of the TRiP stocks with the X chromosome containing this *sc* allele (y^1^ sc^*^ v^1^) revealed the presence of an A>T mutation at position X:1107648 in *sev*, which would result in a premature stop codon at K665X. We tested several of the TRiP stocks that showed the *sev* phenotype using PCR sequencing, and found that all contained the same mutation (Fig. 1C). We have named this new allele *sev^21^*. We note that we observed both *sev^21^* and wild-type flies in BL32421, suggesting that this stock is mixed. Since this premature stop codon would result in a severely truncated protein, it is likely that the *sev^21^* allele would represent a loss-of-function mutation. Supporting this, our newly identified *sev^21^* allele did not complement the known *sev^14^* loss-of-function allele (BL67947, BL32421, BL50662). Since stocks generated by the TRiP at Harvard Medical School and their collaborators at Tsinghua University show the *sev* phenotype, but stocks generated by TRiP collaborators at the National Institute of Genetics in Japan do not, we suspected that the mutation was likely present in the stocks used to balance the TRiP lines (BL35781 and BL32261). PCR sequencing revealed that both of these stocks carry the *sev^21^* allele. Together, these data show that many of the TRiP RNAi stocks balanced with BL35781 or BL32261 contain a newly identified loss-of-function *sev* allele, *sev^21^*. TRiP stocks containing this *sev^21^* allele, including both RNAi and sgRNA lines (TRiP-CRISPR Overexpression and KnockOut) (Port et al. 2014; Jia et al. 2018), will be annotated on Flybase and at BDSC. The presence of the *sev^21^* mutation will not generally affect the use of these stocks, as the X chromosome is typically segregated out or heterozygous during experiments.

## Reagents

BL25709 (RRID:BDSC_25709): y^1^ v^1^ P{y^+t7.7^=nos-phiC31\int.NLS}X; P{y^+t7.7^=CaryP}attP40

BL25710 (RRID:BDSC_25710): y^1^ sc* v^1^ P{y^+t7.7^=nos-phiC31\int.NLS}X; P{y^+t7.7^=CaryP}attP2

BL35781 (RRID:BDSC_35781): y^1^ sc* v^1^; In(2LR)Gla, wg^Gla-1^ PPO1^Bc^/CyO

BL32261 (RRID:BDSC_32261): y^1^ sc* v^1^; Dr^1^ e^1^/TM3, Sb^1^

BL67947 (RRID:BDSC_67947): y^1^ sc* v^1^; P{y^+t7.7^ v^+t1.8^=TRiP.HMS05772}attP40

BL32421 (RRID:BDSC_32421): y^1^ sc* v^1^; P{ y^+t7.7^ v^+t1.8^=TRiP.HMS00416}attP2

BL50662 (RRID:BDSC_50662): y^1^ sc* v^1^; P{ y^+t7.7^ v^+t1.8^=TRiP.HMC03063}attP2

BL42511 (RRID:BDSC_42511): y^1^ v^1^; P{ y^+t7.7^ v^+t1.8^=TRiP.HMJ02076}attP40

BL5690 (RRID:BDSC_5690): sev^14^; Ras85^De2F^/TM3, Sb^1^

BL5691 (RRID:BDSC_5691): sev^14^; drk^e0A^/CyO
